# Cyclic and Acyclic Defensins Inhibit Human Immunodeficiency Virus Type-1 Replication by Different Mechanisms

**DOI:** 10.1371/journal.pone.0009737

**Published:** 2010-03-17

**Authors:** Aprille Seidel, Ying Ye, Lesley R. de Armas, Maira Soto, William Yarosh, Renee A. Marcsisin, Dat Tran, Michael E. Selsted, David Camerini

**Affiliations:** 1 Department of Molecular Biology and Biochemistry, School of Biological Sciences and Center for Virus Research, University of California Irvine, Irvine, California, United States of America; 2 Center for Immunology, University of California Irvine, Irvine, California, United States of America; 3 Department of Pathology and Laboratory Medicine, School of Medicine, University of California Irvine, Irvine, California, United States of America; New York University, United States of America

## Abstract

Defensins are antimicrobial peptides expressed by plants and animals. In mammals there are three subfamilies of defensins, distinguished by structural features: α, β and θ. Alpha and β-defensins are linear peptides with broad anti-microbial activity that are expressed by many mammals including humans. In contrast, θ-defensins are cyclic anti-microbial peptides made by several non-human primates but not humans. All three defensin types have anti-HIV-1 activity, but their mechanisms of action differ. We studied the anti-HIV-1 activity of one defensin from each group, HNP-1 (α), HBD-2 (β) and RTD-1 (θ). We examined how each defensin affected HIV-1 infection and demonstrated that the cyclic defensin RTD-1 inhibited HIV-1 entry, while acyclic HNP-1 and HBD-2 inhibited HIV-1 replication even when added 12 hours post-infection and blocked viral replication after HIV-1 cDNA formation. We further found that all three defensins downmodulated CXCR4. Moreover, RTD-1 inactivated X4 HIV-1, while HNP-1 and HBD-2 inactivated both X4 and R5 HIV-1. The data presented here show that acyclic and cyclic defensins block HIV-1 replication by shared and diverse mechanisms. Moreover, we found that HNP-1 and RTD-1 directly inhibited firefly luciferase enzymatic activity, which may affect the interpretation of previously published data.

## Introduction

Small peptides with antimicrobial, antifungal and antiviral activity have been discovered and classified in various organisms from plants to humans [1], [2], [3]. In humans these peptides are termed defensins. Humans have six α-defensins termed human neutrophil proteins 1 through 4 (HNP 1–4) and human defensins 5 and 6 (HD 5 & 6). HNP 1–4 are made in granulocytes while HD-5–6 are made in Paneth cells, which are found in the crypts of the small intestine [1], [3], [4], [5]. So far four human β-defensins (HBD-1–4) have been characterized although 28 HBD genes have been found in the human genome [6]. No human θ-defensins have been isolated to date, but humans have three θ-defensin pseudogenes that contain premature stop codons. In non-human primates, θ-defensins have been isolated from neutrophils and from bone marrow [7], [8]. Synthetic θ-defensins based on the human pseudogenes have been made *in vitro* and named retrocyclins [9], [10].

The anti-HIV-1 activity of α-defensins has been actively studied since Zhang et al. reported that HNP-1, 2 and 3 were the major components of CD8^+^ cell derived soluble anti-viral factor (CAF) [11]. Subsequent reports found that HNP-1–3 were not produced by CD8 cells, but were present as a result of contamination with neutrophils and/or monocytes [12], [13], [14], [15]. Nevertheless, the anti-HIV-1 activity of HNP-1–3 was confirmed in these studies. Moreover, it has been suggested that HNP-1 blocks HIV-1 replication by inhibiting protein kinase C [16] and α-defensins were found to be upregulated in highly HIV-1 exposed, persistently seronegative individuals [17]. Recently, Furci and colleagues showed that HNP-1 and HNP-2 block viral fusion by binding to the gp120 binding domain of CD4 [18]. Our data support these mechanisms of α-defensin inhibition of HIV-1 and suggest that other mechanisms may also be operative.

The β-defensins HBD-1, 2 and 3 have been shown to have anti-HIV-1 activity *in vitro* at concentrations that are present *in vivo* [19], [20]. Further evidence that β-defensins are important in defense against HIV-1 *in vivo* comes from a study in which a polymorphism in the HBD-1 gene was associated with HIV-1 infection in a population of children [21]. Beta-defensins-1 and 2 have also been found to be upregulated in the alveolar macrophages of HIV-1 positive individuals [22] and both HBD-2 and HBD-3 specifically downregulate cell-surface expression of CXCR4 [19], [23]. Moreover, β-defensins may be responsible in part for protection from HIV-1 transmission in the oral cavity [19], [20], [24]. Both α- and β-defensins have been found in human breast milk and have been shown to decrease mother to infant transmission of HIV-1 [25], [26], [27], [28]. Furthermore, HBD-3 and the θ−defensin RC2 were shown to block influenza viral fusion by cross-linking cell surface glycoproteins [29], a mechanism that may confer broad-spectrum antiviral activity.

Theta-defensins are the only known circular peptides in the animal kingdom, but to date they have only been isolated from non-human primates [7], [8]. Naturally occurring and synthetic θ−defensins have anti-HIV-1 activity [9], [10], [30], [31], [32]. Moreover, Owen *et al* found that synthetic θ−defensins were effective against primary isolates of HIV-1 [33], [34]. Theta-defensins have also been shown to have activity against herpes simplex virus [35]. Since humans encode θ−defensin pseudogenes, but do not produce θ−defensins, highly HIV-1 exposed persistently seronegative individuals were examined for changes in these pseudogenes, but none were found in one study [36]. It has been proposed that θ−defensins are lectins and that their anti-viral activity occurs at the level of entry perhaps by blocking virus-receptor interactions [10], [29], [32], [35]. Both α-defensins and θ-defensins have been shown to bind gp120 and CD4 [18], [37]. Recent studies have shown that θ-defensins are able to interact with viral gp41 to prevent HIV-1 *env* mediated fusion with the cytoplasmic membrane by blocking the formation of the six-helix bundle required for viral fusion [30], [31].

Alpha-, β- and θ-defensins all show anti-viral activity against CCR5 tropic (R5) and CXCR4 tropic (X4) HIV-1. Here we examined the mechanisms of action of one prototypical defensin in each of the three subfamilies against both R5 and X4 HIV-1. HNP-1, HBD-2 and rhesus θ−defensin-1 (RTD-1) were chosen because they have activity against HIV-1 and because they are among the most abundant defensins in their respective classes *in vivo*. We tested the anti-HIV-1 activity of each defensin when added to cells at various times pre- and post-infection and investigated their effect on HIV-1 entry and reverse transcription to distinguish between inhibition of early and late steps in viral replication. In addition, we examined the ability of HNP-1, HBD-2 and RTD-1 to downmodulate CD4, CCR5 and CXCR4 expression and to directly inactivate HIV-1 virions or HIV-1 derived vector particles.

## Results

### HNP-1 and RTD-1 inhibit luciferase activity

**Figure 1:**
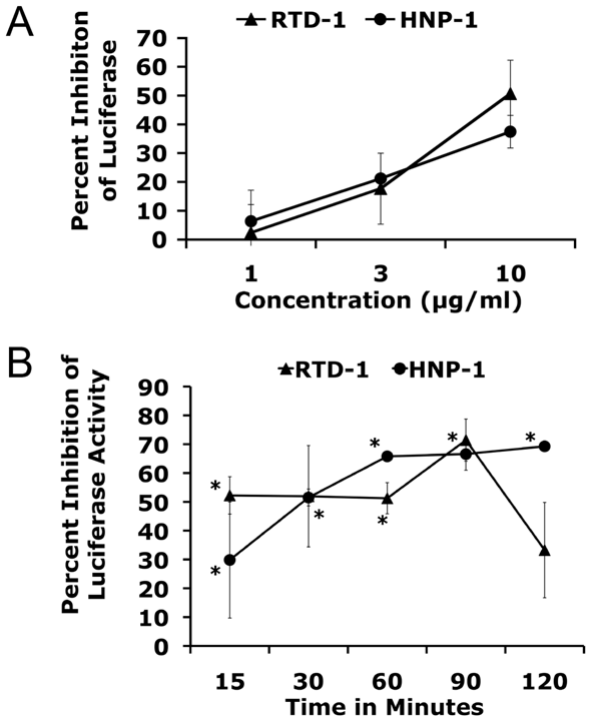
HNP-1 and RTD-1 inhibited luciferase activity in a concentration dependent manner. (A) 293T cells transfected with CDM8-luc were lysed 48 hours post transfection and then cell lysates were incubated with HNP-1 or RTD-1, at 0, 1, 3, or 10 µg/ml on ice for 1 hour and luciferase activity was measured. (B) HNP-1 and RTD-1 were added to lysates of 293T cells transfected with CDM8-luc. Cell lysates were incubated with 10 µg/ml HNP-1 or RTD-1 on ice for 15, 30, 60, 90, or 120 minutes and luciferase activity was measured. The data points in (A) and (B) represent triplicate samples from two experiments. Error bars show standard deviations from the mean. Asterisks represent p<0.01 by Student's t-test compared to buffer only.

Previous studies on defensin activity against HIV-1 have employed luciferase reporter constructs to monitor HIV-1 replication [12], [16], [32], [33], [34], [37]. We found that firefly luciferase activity in a cell lysate was significantly diminished, in a concentration dependent manner, in the presence of HNP-1 or RTD-1 (Fig. 1A). This inhibition was manifest with incubation times as short as 15 minutes in the presence of 10 µg/ml HNP-1 or RTD-1 (Fig. 1B). In contrast HBD-2 had no significant effect on luciferase activity at the same range of concentrations and incubation times (data not shown). Our results indicate that quantitation of defensin-mediated inhibition of HIV-1 infection by luciferase assay may be unreliable since α- and θ−defensins directly antagonize luciferase enzymatic activity. We also tested the activity of all three defensins on GFP expression and HIV-1 capsid protein (p24) ELISA. None of the three defensins tested inhibited GFP or p24 detection (data not shown). In our studies, Tat-dependent expression of GFP in GHOST cells and p24 ELISA of infected peripheral blood mononuclear cells (PBMC) were used to evaluate defensin inhibition of HIV-1.

### HNP-1, HBD-2 and RTD-1 inhibit HIV-1 replication in a dose-dependent manner, but are not cytotoxic

**Figure 2:**
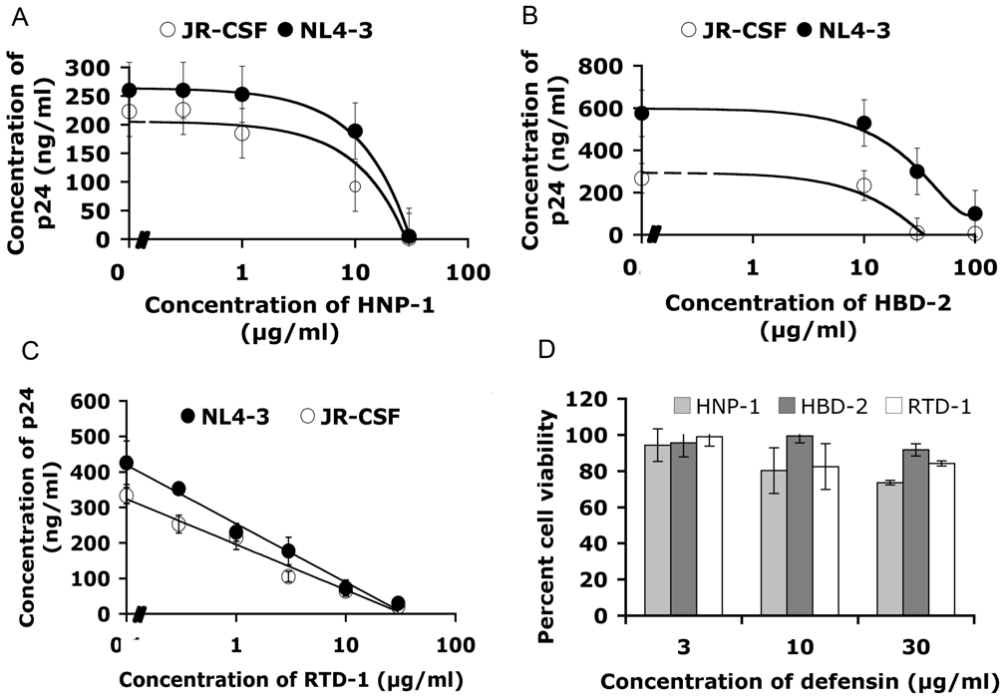
HNP-1, HBD-2 and RTD-1 inhibited HIV-1 replication in PBMC. PBMC were incubated for one hour with 0, 3, 10 or 30 µg/ml HNP-1 (A), HBD-2 (B) or RTD-1 (C) and then infected with either X4 HIV-1 (NL4-3; solid symbols) or R5 HIV-1 (JR-CSF; open symbols) both at an MOI of 0.1. Three days post infection cell culture supernatants were collected and analyzed for HIV-1 capsid, p24, by ELISA. Data shown for HNP-1 and HBD-2 are from three experiments done in triplicate and data for RTD-1 are from two experiments done in triplicate. Error bars show standard deviations from the mean. (D) To test the cytotoxicity of defensins, PBMC were incubated with HNP-1, HBD-2 or RTD-1 at 3, 10 or 30 µg/ml for two days and then formation of colored formazan products after addition of MTS was measured at 490 nm on a spectrophotometer. Data are from a single experiment done in triplicate and normalized to untreated cells. Error bars show standard deviations from the mean. No statistically significant changes in cell viability were detected (p<0.05).

In order to determine the anti-HIV-1 potency of HNP-1, HBD-2 and RTD-1, increasing concentrations of HNP-1, HBD-2 or RTD-1 were added to PBMC one hour prior to infection with R5 or X4 HIV-1 at a multiplicity of infection (MOI) of 0.1. Inhibition of HIV-1 replication was monitored by determining the amount of viral p24 in tissue culture supernatants three days post-infection. PBMC treated with each of the three defensins showed a dose-dependent inhibition of virus replication in comparison with control cells (Fig. 2A–C). The calculated IC_50_ values for HNP-1 are 8 µg/ml (2.3 µM) and 9 µg/ml (2.6 µM), for HBD-2, 30 µg/ml (6.9 µM) and 25 µg/ml (5.8 µM) and for RTD-1, 7.2 µg/ml (3.5 µM) and 7.5 µg/ml (3.6 µM) respectively for inhibition of X4 and R5 HIV-1 replication. In parallel with the anti-HIV-1 assay, we measured the metabolic activity of cells treated with different amount of HNP-1, HBD-2 or RTD-1. None of the defensins caused significant cell death at active anti-viral concentrations when measured by metabolic formazan production in an MTS assay (Fig. 2D).

### HNP-1, HBD-2 and RTD-1 inhibit HIV-1 replication at different stages

**Figure 3:**
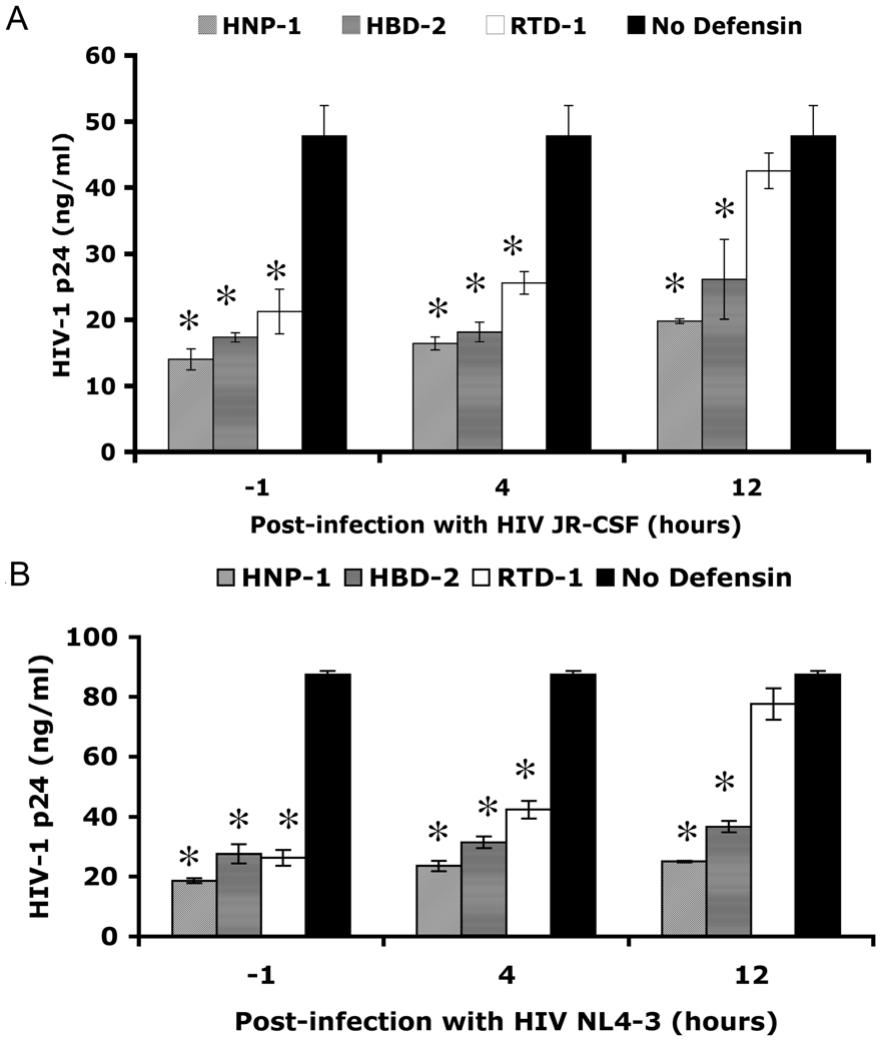
HNP-1, HBD-2 and RTD-1 inhibited HIV-1 replication depending on the time of addition. HNP-1, HBD-2 or RTD-1 (10 µg/ml) were added to PHA-stimulated PBMC one hour prior to infection or four or twelve hours after infection with JR-CSF (A) or NL4-3 (B) in triplicate wells, at an MOI of 0.1. The results shown are from p24 ELISA of the media at seventy-two hours post infection. Error bars show the standard deviations of triplicate samples. Asterisks denote significant differences from the no defensin control by Student's t-test (p<0.01). The results shown are representative of three experiments performed in triplicate.

To determine whether HNP-1, HBD-2 or RTD-1 inhibited viral entry or later steps in HIV-1 replication, activated PBMC were infected with R5 or X4 HIV-1 (MOI = 0.1) and HNP-1, HBD-2 or RTD-1 were added one hour prior to infection or up to 12 hours post infection and viral replication was measured 72 hours post infection (Fig. 3). HNP-1 was able to inhibit both X4 and R5 HIV-1 replication when added prior to infection or when added up to 12 hours after infection. Similarly, HBD-2 inhibited X4 and R5 HIV-1 replication even when added 12 hours after infection but to a lesser degree than HNP-1. Both HNP-1 and HBD-2 therefore were able to inhibit HIV-1 replication after viral entry although a small amount of entry inhibition was also evident, particularly for HBD-2. In contrast, RTD-1 only inhibited HIV-1 replication when present at the time of infection or four hours post infection when entry was likely still occurring. When RTD-1 was added 12 hours after infection no significant inhibition of HIV-1 replication was detected, indicating that RTD-1 inhibited HIV-1 entry or an early step in viral replication in this assay.

### HIV-1 entry is significantly inhibited by RTD-1 but not by HNP-1 or HBD-2

**Figure 4:**
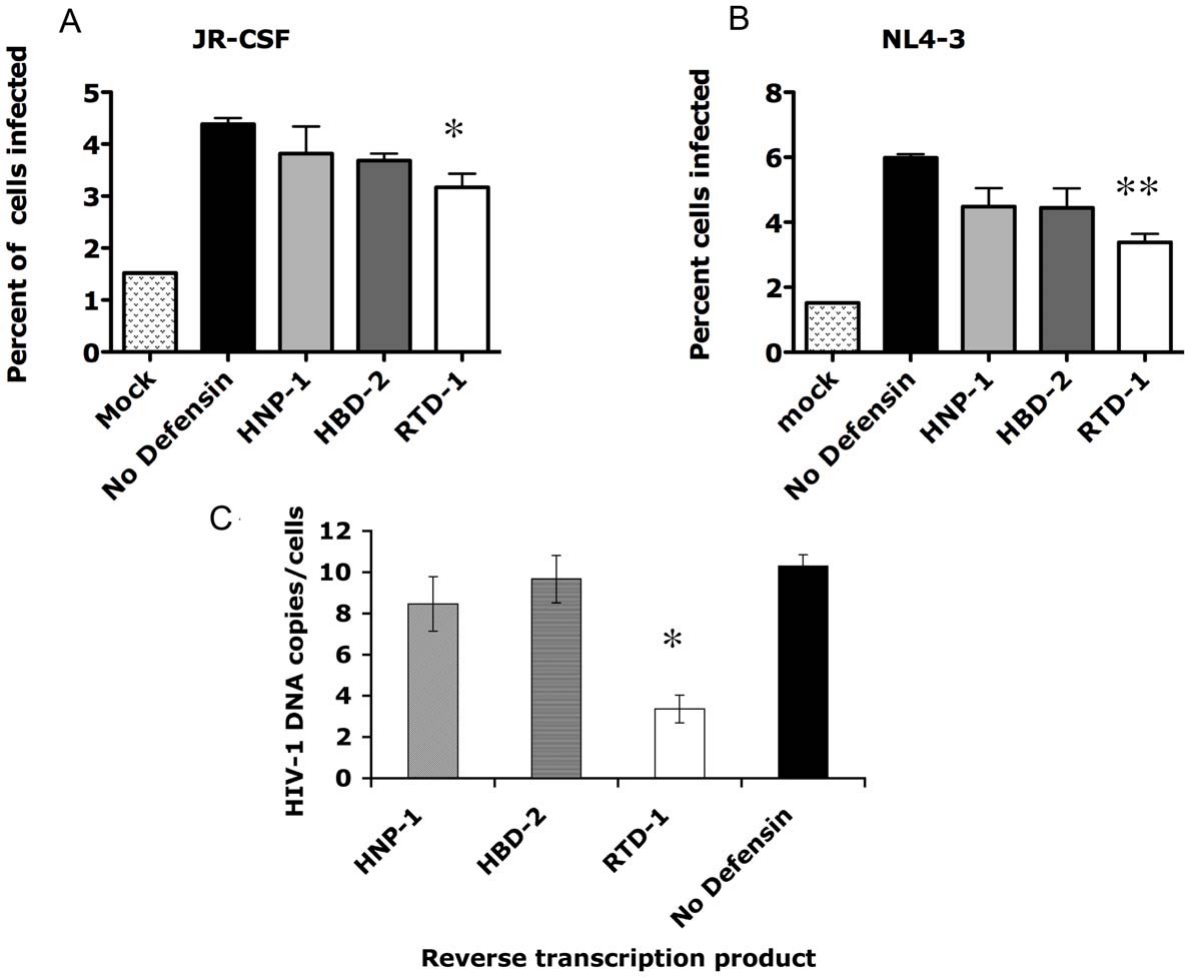
Defensins inhibit viral fusion at different levels. Viral fusion was inhibited by the addition of defensins for JR-CSF (A) and NL4-3 (B) virus. Using ANOVA for comparison single asterisk is p<0.05 and double asterisks is p<0.01. Only RTD-1 significantly reduced viral fusion. Panel C. RTD-1 blocked HIV-1 reverse transcription, but HNP-1 and HBD-2 did not. HNP-1, HBD-2 or RTD-1 (10 µg/ml) were added to PHA-stimulated PBMC and incubated for one hour, followed by infection with HIV-1 (JR-CSF at an MOI of 0.1). Forty-eight hours later the cells were lysed and DNA was isolated for real-time quantitative PCR to evaluate the synthesis of HIV-1 cDNA. The primers M667 and M661 were chosen to detect complete viral cDNA. Copies of HIV-1 DNA were normalized against copies of beta-globin. Error bars indicate the standard deviations of triplicate samples. The results shown are representative of two experiments performed in triplicate. The asterisk denote p<0.05 by Student's t-test compared to the no defensin control.

We used an enzyme-based flow cytometric HIV-1 entry assay developed by Cavrois *et al* to measure inhibition of HIV-1 entry by HNP-1, HBD-2 and RTD-1 [38]. The assay utilized a β-lactamase-Vpr (BlaM-Vpr) fusion protein that was incorporated into virions. Target cells were loaded with CCF2, whose fluorescence emission is changed from 520 nm to 447 nm upon cleavage of a β-lactam moiety. Entry of HIV-1 virions bearing β-lactamase-Vpr fusion protein into target cells containing CCF2 was detected by an increase in fluorescence at 447 nm. The virtue of the assay is that it is specific for viral entry into cells but the fluorescent signal is relatively weak so the percent of cells infected was likely underestimated. All three defensins inhibited R5 and X4 HIV-1 entry into stimulated PBMC to some degree when present at 10 µg/ml (Fig. 4A and 4B). Only RTD-1, however, inhibited HIV-1 entry in a statistically significant manner in this assay.

### HIV-1 replication is inhibited prior to reverse transcription by RTD-1 and after reverse transcription by HNP-1 and HBD-2

To further determine the stage at which HIV-1 replication is inhibited by HNP-1, HBD-2 and RTD-1, we added 10 µg/ml of each of the three defensins to PHA-stimulated PBMC and incubated for one hour, then infected the cells with R5 HIV-1 (MOI = 0.1), and harvested them 48 hours later. The cells were lysed and DNA was isolated to evaluate the synthesis of HIV-1 cDNA by real-time quantitative PCR. The LTR-R region and packaging site primers M667 and M661 were chosen to detect completed HIV-1 cDNA molecules. Copies of HIV-1 were normalized against copies of β−globin to control for differences in cell number between samples. RTD-1 blocked HIV-1 cDNA formation, while HNP-1 and HBD-2 did not significantly affect the level of HIV-1 cDNA molecules in HIV-1 infected cells (Fig. 4C). This difference implies that HNP-1 and HBD-2 inhibited HIV-1 replication after reverse transcription was completed, but RTD-1 inhibited HIV-1 replication prior to or during reverse transcription.

### HNP-1, HBD-2 and RTD-1 downmodulate the chemokine receptor CXCR4

**Figure 5:**
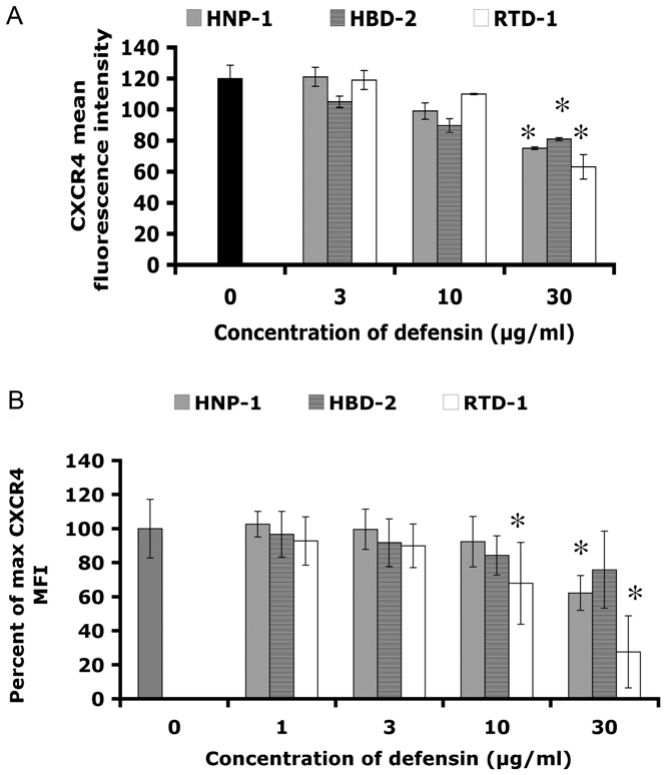
HNP-1, HBD-2 and RTD-1 cause down modulation of CXCR4. (A) HNP-1, HBD-2 or RTD-1 were incubated with GHOST-R5X4 cells, at 0, 3, 10 or 30 µg/ml for 3 hours at 37°C in Iscove's media without serum. The cells were then stained with an anti-CXCR4-PE MAb and analyzed by flow cytometry. Data represent the average of two experiments performed in triplicate. (B) PBMCs were incubated with 0, 3, 10 or 30 µg/ml of HNP-1, HBD-2 or RTD-1 for 3 hours at 37°C in Iscove's media without serum. Two monoclonal antibodies against CXCR4 were used to stain the cells to be analyzed by flow cytometry in different experiments. Results from six assays, three for each antibody, are shown as a percentage of untreated samples. Error bars show standard deviations from the mean. Asterisks represent p<0.05 by Student's t-test compared to the no defensin control.

We assayed the ability of each defensin to downmodulate CD4, CCR5 and CXCR4. GHOST-R5X4 cells or PBMC were incubated for three hours in serum free media with 0, 3, 10 or 30 µg/ml HNP-1, HBD-2 or RTD-1 at 37°C or on ice. Cells were then washed and incubated with CD4-PerCP, anti-CCR5-APC and anti-CXCR4-PE monoclonal antibodies. The mean fluorescence intensity in each channel was measured by flow cytometry. HNP-1, HBD-2 and RTD-1 all downmodulated CXCR4 expression in a dose dependent manner in both GHOST-R5X4 cells (Fig. 5A) as well as in PBMC (Fig. 5B) only when incubated at 37°C. In contrast, there was no significant downmodulation of cell surface expression of CD4 or CCR5 with any of the three defensins (data not shown). Incubation of GHOST-R5X4 cells or PBMC with defensins on ice did not alter detection of CD4, CCR5 or CXCR4 indicating that antibody binding was not blocked by defensins.

### Defensins inactivate HIV-1

**Figure 6:**
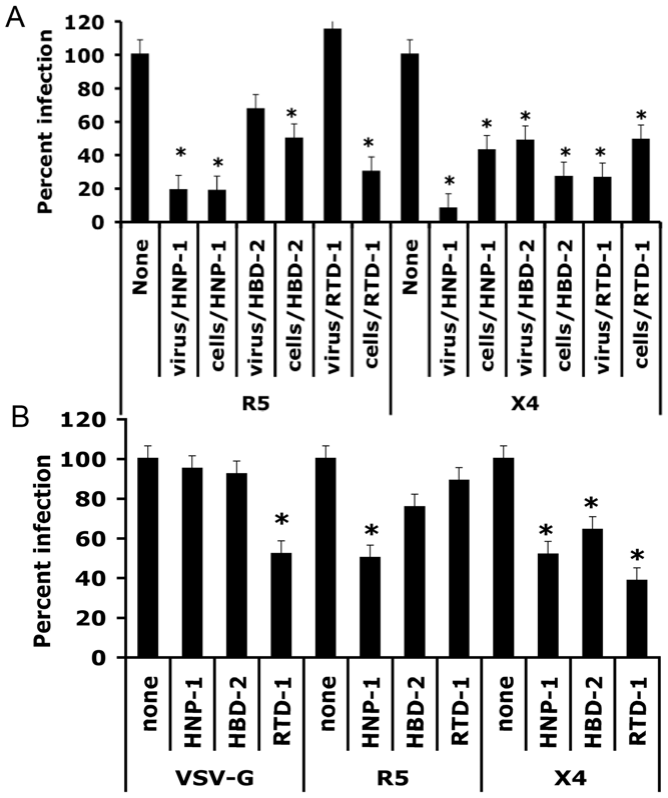
HIV-1 inactivation by HNP-1, HBD-2 and RTD-1. (A) JR-CSF (R5 HIV-1) or NL4-3 (X4 HIV-1) sufficient for an MOI of 0.1 were incubated with 10 µg/ml HNP-1, HBD-2, or RTD-1 for one hour on ice. The virus and defensin mixture was then centrifuged over a Sephadex G-25 column to remove defensin. The defensin-free virus was then added to PBMC or GHOST-R5X4 cells (labeled in graph as virus/defensin). Alternatively, defensin was incubated with PHA-stimulated PBMC or GHOST-R5X4 cells for one hour at 37°C and washed away prior to the time of infection at an MOI of 0.1 with JR-CSF or NL4-3 (labeled in graph as cells/defensin). PBMC supernatants were collected three days after infection and analyzed by ELISA for the presence of viral capsid, p24. GHOST-R5X4 cells were harvested 36 hours after infection and analyzed by flow cytometry for GFP expression. PBMC were used for HNP-1 and HBD-2 assays while GHOST-R5X4 cells were used for RTD-1 assays. Values were normalized to show percent infection compared to the uninhibited control. Data shown are the average of three experiments done in triplicate. Error bars represent the standard deviation from the mean. Asterisks represent p<0.05 calculated using Student's t-test. (B) Lentiviral vectors pseudotyped with VSV-G, R5 or X4 HIV-1 envelope were incubated with 10 µg/ml HNP-1, HBD-2, or RTD-1 for one hour on ice. The viral vector and defensin mixture was then centrifuged over a Sephadex G-25 column to remove defensin. The defensin free vector was then added to GHOST-R5X4 cells at an MOI of 0.1. Cells were harvested thirty-six hours after infection and GFP expression was analyzed by flow cytometry compared to the uninhibited control. Data shown are the average of four experiments done in triplicate; error bars represent the standard deviation from the mean. Asterisks represent p<0.05 calculated using Student's t-test.

To address whether defensins act only on cells or also inhibit infection by interacting directly with HIV-1, we performed virus inactivation studies. R5 or X4 HIV-1 was incubated for one hour on ice with HNP-1, HBD-2 or RTD-1 in serum free media and then the virus and defensin were separated on a G-25 Sephadex column. Virus collected from the columns was incubated with activated PBMC or GHOST-R5/X4 cells. PBMC were then incubated 48 hours and the supernatants were collected for p24 ELISA. GHOST cells were collected 36 hours post infection for flow cytometric analysis of GFP expression. For comparison, cells were incubated for one hour with HNP-1, HBD-2 or RTD-1 and then washed prior to infection with virus that was not incubated with defensin. As a control for loss of viral infectivity during the experimental protocol, cells were infected with R5 or X4 HIV-1 that was incubated for one hour on ice in the absence of defensin and then eluted from a G-25 column (mock treatment, labeled “None” in Fig. 6A and 6B). Statistical significance was determined by a two-tailed Student's t-test. HNP-1 significantly inactivated both R5 and X4 HIV-1 in this assay. HBD-2 also inactivated both X4 and R5 HIV-1, but only the X4 HIV-1 replication was inhibited to a statistically significant level. In contrast, RTD-1 inactivated X4 HIV-1, but did not inactivate R5 HIV-1.

We generated envelope pseudotyped lentiviral vectors which express HIV-1 Tat and GFP and tested the ability of HNP-1, HBD-2 or RTD-1 to inactivate the pseudotyped HIV-1 based vectors. Vesicular stomatitis virus glycoprotein (VSV-G), X4 HIV-1 and R5 HIV-1 envelopes were used to pseudotype the vectors. HNP-1, HBD-2 or RTD-1 were incubated with the pseudotyped vectors for one hour on ice and then defensin and lentiviral vectors were separated on a G-25 Sephadex column as described above. The pseudotyped vectors collected from the column were then used to infect GHOST-R5X4 cells. Cellular GFP expression was analyzed by flow cytometry 48 hours later (Fig. 6B). HNP-1 and HBD-2 did not inactivate VSV-G pseudotyped vectors, but did inactivate X4 and R5 HIV-1 Env pseudotyped vectors. Inhibition of the R5 pseudotyped vector by HBD-2, however, was not statistically significant by a two-tailed Student's T test. RTD-1 was able to inactivate VSV-G and X4 HIV-1 pseudotyped vectors, but did not inactivate R5 HIV-1 pseudotyped vector. This finding is consistent with the inactivation of X4 but not R5 HIV-1 strains by RTD-1 shown in Fig. 6A.

## Discussion

The roles of α-, β- and θ-defensins in antiviral innate immunity have been analyzed in several recent studies [12], [16], [29], [32], [39], [40] a number of which relied on luciferase-based reporter assays. As shown here, some defensins are potent inhibitors of firefly luciferase, thereby complicating attempts to quantify antiviral activity using luciferase reporter assays. To circumvent these limitations, we used an HIV-1 capsid protein (p24) ELISA of infected PBMC or GFP expression from an indicator cell line (GHOST cells) to test the anti-HIV-1 activity of HNP-1, HBD-2, and RTD-1.

HNP-1 and HBD-2 significantly inhibited HIV-1 replication at concentrations near or below their reported physiological concentrations in human breast milk (∼10 µM) and they may therefore limit vertical transmission of HIV-1 via this route [41]. In contrast, the concentration of HNP-1 in plasma has been reported to be at least an order of magnitude lower than the IC_50_ against HIV-1 that we determined, so HNP-1 is unlikely to be effective against HIV-1 in plasma [42]. The total concentration of HNP-1 in the circulation, however, including the amount contained within leukocytes and in plasma, is sufficient to inhibit HIV-1 [42]. Moreover, defensin concentrations encountered by HIV-1 in physiologic microenvironments within phagocytic cells, or in extracellular spaces, may be substantially higher. Similarly, HBD-2 is found at approximately 10 µM in oral tissue [43], and its mRNA is induced by HIV-1 infection [19], so HBD-2 and other β-defensins are likely effective against HIV-1 in the oral cavity and may contribute to preventing oral transmission. Like HNP-1 in humans, RTD-1 is found in rhesus macaque neutrophils at sufficient concentration to inactivate HIV-1 [7]. RTD-1 and related θ-defensins may therefore play a role in innate immunity to SIV in rhesus macaques and in other non-human primate species including baboons if their activity against the relevant SIV is similar to their activity against HIV-1.

HNP-1 and HBD-2 may inhibit HIV-1 replication by more than one mechanism *in vivo*. Both defensins directly inactivated R5 and X4 HIV-1 when incubated with virus and both HNP-1 and HBD-2 inhibited HIV-1 replication when added to cells many hours after the initiation of HIV-1 infection. When added to cells, HNP-1 and HBD-2 inhibited virion production after reverse transcription was complete. These results suggest at least two mechanisms of inhibition. Chang *et al* published similar results for HNP-1 in a different cell type [16]. Moreover, the results of Quinones-Mateu *et al* with HBD-2 are consistent with our data except that they found R5 HIV-1 to be less sensitive to HBD-2 than X4 HIV-1, perhaps because they used oral epithelial cells while we used PBMC [16].

RTD-1 may also inhibit HIV-1 replication by more than one mechanism, but in contrast to HNP-1 and HBD-2, RTD-1 inactivated X4 but not R5 HIV-1 and inhibited HIV-1 replication only when added to cells prior to, at, or near the time of infection. Moreover, RTD-1 significantly inhibited HIV-1 entry while HNP-1 and HBD-2 did not. These results imply that at least one of RTD-1's mechanisms of action is different from those of HNP-1 and HBD-2. Furthermore, our results with RTD-1 are consistent with the results of others showing that θ−defensins bind the HIV-1 surface glycoprotein gp120, as well as the HIV-1 receptor CD4 and block HIV-1 entry into cells [10], [29], [32], [35].

The acyclic α- and β-defensins, HNP-1 and HBD-2 respectively, were able to inactivate both X4 and R5 HIV-1 strains, while the cyclic θ-defensin, RTD-1, only inactivated X4 HIV-1. This result was found to hold true for pseudotyped lentiviral vectors as well as replication competent HIV-1, although inhibition of R5 HIV-1 and vector by HBD-2 did not reach significance. RTD-1 was also able to inactivate VSV-G pseudotyped vectors, while the linear defensins could not.

Defensins have been reported to bind to glycoproteins including CD4, and gp120 as well as polysaccharides and glycans [10], [29], [32]. Moreover, CXCR4 was shown to be internalized and surface expression downregulated following β-defensin binding [19], [23], [44]. GHOST cells uniformly expressing CD4, CCR5 and CXCR4 (80%, 89% and 90% positive respectively) as well as PBMC were incubated with HNP-1 HBD-2, or RTD-1 for three hours and then CD4, CCR5 and CXCR4 expression was analyzed by flow cytometry. All three defensins downmodulated the expression of CXCR4 in a dose-dependent manner in both types of cells, but no significant effect on the expression of CD4 or CCR5 was observed. Our data are in agreement with and extend previous studies that used PBMC and a similar protocol. Down modulation of cell surface CXCR4 needed by X4 HIV-1 to enter the cell is therefore another possible mechanism of inhibition of X4 HIV-1 by defensins. This is likely not the primary mechanism of HIV-1 inhibition by HNP-1, HBD-2 or RTD-1, however, since relatively high levels of defensin are needed for CXCR4 down modulation and since R5 HIV-1 replication was inhibited as well as X4 HIV-1 replication.

In summary, HNP-1, HBD-2 and RTD-1 are potent inhibitors of HIV-1 replication at physiological concentrations. Both acyclic and cyclic defensins likely inhibit HIV replication by multiple mechanisms. For therapeutic use, defensins may have a role as topical virucides to prevent transmission. Moreover, boosting α- and β-defensin responses to HIV-1 infection in patients may help slow the course of disease progression. Continuing studies should seek to define the mechanisms of defensin mediated inhibition of HIV-1 replication, which may guide their use as potential prophylactics or therapeutics and will increase our knowledge of innate immunity to HIV-1 infection. Moreover, future studies should include diverse primary HIV-1 isolates from all major clades to assess the generality and clinical utility of defensins.

## Materials and Methods

### Defensins

HNP-1 was purified from human neutrophils as previously reported [45], [46]. The purity of the peptide was confirmed by reversed-phase high performance liquid chromatography (HPLC) and acid–urea polyacrylamide gel electrophoresis (PAGE). A synthetic version of RTD-1 was produced by solid-phase synthesis as previously described [7]. HBD-2 was purchased from Chemicon International. (Temecula, CA). All defensin stocks were 1 mg/ml in 10 mM acetic acid.

### HIV-1 stocks

Plasmids pNL4-3 and πSV-JR-CSF bearing full-length infectious molecular clones of HIV-1 NL4-3 and JR-CSF respectively were used to transfect 293T cells (obtained from ATCC) by calcium phosphate transfection. Briefly, 30 µg of plasmid DNA was mixed with 2 M CaCl_2_ and 10 X NTE buffer then added to an equal volume of 2 X HEPES and phosphate buffered saline. The resulting DNA-calcium phosphate coprecipitate was then incubated with 293T cells in the presence of 25 mM chloroquine for five hours and then washed away. Media was changed 24 hours later to Iscove's complete media with 2% fetal bovine serum and virus from transfected cells was collected on day three or four post transfection. Virus stocks were titered for seven days on PHA activated PBMC and infection was monitored by p24 ELISA as previously described [47]. HIV-1 stocks were used to infect cells at an MOI of 0.1.

### Purification of PBMC

Whole blood was collected with written informed consent from healthy donors at the UCI blood bank or at the UCI General Clinical Research Center. Whole blood or enriched leukocytes were mixed one to one with PBS and then layered over ficoll-hypaque. PBMC were isolated by centrifugation for thirty minutes at 1150 RPM, then removed and washed in PBS. Red blood cells (RBC) were lysed by suspension in 0.8% NH_4_Cl with 0.1 mM EDTA. After RBC lysis, PBMC were washed twice in PBS and then cultured in RPMI supplemented with 10% FBS and 4 µg/ml PHA. After three days PHA was removed from the media.

### Luciferase assay

293T cells were transfected by calcium phosphate co-precipitation as described above with CDM8-*luc* plasmid. After five hours media was aspirated and replaced with fresh media. Thirty-six or 48 hours later 293T cells were lysed according to the manufacturer's protocol, using a Promega (Madison, WI) luciferase assay system. Lysates were incubated on ice for one hour with 1, 3 or 10 µg/ml of HNP-1. HBD-2, or RTD-1 and then luciferase activity was measured with a luminometer. Alternatively, 293T CDM8-*luc* lysates were incubated with 10 µg/ml of HNP-1 or RTD-1 for 15, 30, 60, 90 or 120 minutes and then luciferase activity was measured by luminometer. 293T cells were maintained in Iscove's modified Dulbecco's media (IMDM) supplemented with 10% fetal bovine serum (FBS), 200 mM L-glutamine, 50 µg/ml gentamicin, and 500 µg/ml G418. During and after transfection, Iscove's media without FBS or G418 was used.

### Determination of inhibition of HIV-1 replication

PBMC were washed free of media containing fetal bovine serum (FBS), then infected with NL4-3 or JR-CSF at an MOI of 0.1 in the presence of varying concentrations of HNP-1, HBD-2 or RTD-1 by centrifugation for ninety minutes at 2500 RPM and 25°C (spinfection) in RPMI media without serum followed by culture in RPMI supplemented with 5% FBS [48]. Supernatants from PBMC were harvested three days post infection and analyzed by p24 ELISA. For the time of addition studies, HNP-1, HBD-2 or RTD-1 were added to activated PBMC one hour prior to infection at an MOI of 0.1 or at various times post infection. PBMC were washed free of media containing FBS and resuspended in RPMI only, defensin was added to some wells. Virus was added to all wells and the plates were spinfected. Cells were washed twice in PBS and re-plated in RPMI supplemented with 5% FBS. HNP-1, HBD-2 or RTD-1 were added back to the wells that were preincubated with each, or added at various times post infection. Seventy-two hours later, the supernatant was harvested and assayed for p24 by ELISA.

### p24 ELISA

p24 ELISA kits were purchased from Perkin Elmer (Boston, MA). The assay was performed according to the manufacturer's protocol. Alternatively p24 expression levels were determined by an ELISA protocol using a p24 monoclonal antibody (183-H12-5C) and pooled human anti-HIV immunoglobulin G [49]. The p24 antibody was obtained from the AIDS Research and Reference Reagent Program and was contributed by Bruce Chesebro. Samples were diluted 1∶100 at the time of assay. To test the effects of defensins on the ELISA, HIV-1 capsid protein, p24, was diluted to 400, 200, or 100 pg/ml. After two hours of incubation, ELISA plates were washed and incubated with biotin-labeled pooled human anti-HIV-1 immunoglobulin G. After one hour incubation and washing, streptavidin-peroxidase was added followed by o-phenylenediamine dihydrochloride (OPD) to obtain a color change. Sulfuric acid was added to a final concentration of 220 mM to stop the reaction and assay results were read at 490 nm with a reference filter at 610 nm.

### Cell viability

Cell viability was tested using the Promega (Madison, WI) MTS assay according to the manufacturer's protocol. Briefly, PBMC were incubated for 48 hours with defensins in media without FBS, subsequently the cells were assayed for viability. MTS is a tetrazolium salt that is bioreduced to a formazan product in living cells. MTS (2 ml) was mixed with phenazine methosulfate (100 µl) and then 20 µl of the mixture was added to wells of 96 well plates each containing 10^6^ cells in 100 µl media. The plate was then incubated for 3 hours and read on a spectrophotometer at 490 nm. Formazan production in cells treated with defensins was normalized to that of untreated cells.

### HIV-1 entry assay

The BlaM-Vpr fusion assay was carried out as previously described [38]. Briefly, one hour prior to infection PHA stimulated PBMC were incubated with 10 µg/ml of either HNP-1, HBD-2 or RTD-1 or with media alone. After 1 hour at 37°C the PBMC were infected with either X4 HIV-1-BlaM-Vpr or R5-HIV-1-BlaM-Vpr for 2 hours at 37°C. Subsequently, the PBMC were washed in Iscove's medium and loaded with CCF2-AM (Invitrogen) according to the manufacturer's protocol. After loading, the cells were incubated for one hour at room temperature in Iscove's medium containing 10% FBS. Cells were then washed in PBS and resuspended in PBS containing 2% paraformaldehyde for flow cytometric analysis. Cells were analyzed using an LSR-II flow cytometer and FlowJo software.

### Quantitative real-time PCR

HNP-1, HBD-2 or RTD-1 was added to PHA-stimulated PBMC at 10 µg/ml and incubated for one hour. Next, the PBMC were infected with HIV-1 (JR-CSF) by spinfection [48], then 48 hours later 3.0×10^6^ cells were lysed in 100 µl of 100 µg/ml proteinase K in 10 mM Tris–HCl, pH 8.0 at 56°C for 1 hour, followed by heat inactivation at 95°C for 10 min. Each PCR reaction contained 15 µl SYBR green PCR master mix (Applied Biosystems), 5 µl of cell lysate, 0.3 mM of each primer in a 30 µl reaction volume. HIV-1 specific primers M661, CCTGCGTCGAGAGAGAGCTCCTCTGG (nts 695–672) and M667, GGCTAACTAGGGAACCCACTG (nts 496–516) were used to detect complete cDNA. Copies of HIV-1 DNA were normalized against copies of β-globin using primers LA1, ACACAACTGTGTTCACTAGC and LA2, CAACTTCATCCACGTTCACC directed to β-globin.

### Receptor down modulation studies

GHOST-R5X4 cells (obtained from NIH AIDS Research & Reference Reagent Program) were trypsinized, washed and counted, then 5×10^5^ cells were placed into microfuge tubes in IMDM without FBS and without G418. Cells were incubated with 0, 3, 10, or 30 µg/ml of HNP-1, HBD-2 or RTD-1 for 3 hours at 37°C or on ice. Cells were washed twice with PBS + 0.02% sodium azide and stained with monoclonal antibodies to CXCR4 (clone 12G5-PE, BD Pharmingen, San Diego, CA), CCR5 (clone 3A9-APC, R & D systems, Minneapolis, MN) and CD4-PerCP (BD Pharmingen, San Diego, CA). Cells were analyzed by 3-color flow cytometry. Ghost cells were maintained in IMDM supplemented with 10% FBS, 200 mM L-glutamine, 50 µg/ml gentamicin, and 500 µg/ml G418. Receptor downmodulation studies on PBMC were performed with freshly purified samples. PBMC were plated in a 96 well plate at 5×10^5^ cells/well in IMDM without any additives. HNP-1, HBD-2 or RTD-1 were added to a final concentration of 0, 3 10 or 30 µg/ml. After a three hour incubation with defensins at either 37°C or 4°C, cells were washed three times with PBS + 2% FBS. Monoclonal antibodies against CXCR4 (clone 12G5-PE, CalTag, Burlingame, CA or clone 12G5-biotin, BD Pharmingen, San Diego, CA), CCR5 (clone 3A9-APC, R & D systems, Minneapolis, MN) and CD4 (clone S3.5-Alexa 488, CalTag, Burlingame, CA) were used to measure receptor expression. Streptavidin-PE (CalTag, Burlingame, CA) was also used to visualize CXCR4 expression when the biotin conjugated antibody was used. Stained cells were then analyzed by 3-color flow cytometry. Since two different antibodies were used to stain CXCR4 the results were normalized and are shown as a percentage of the mean fluorescence intensity (MFI) of untreated cells.

### Generation of pseudotyped lentiviral vectors

Pseudotyped HIV-1 derived vectors were made by CaPO_4_ triple transfection of 293T cells with a packaging plasmid (pCMVΔR8.2), a transfer vector (pHR-Tat-IRES-eGFP) and an envelope plasmid (pHCMV-VSV-G or pCMV-JR-CSF*env* (32D2 V1–V3) or pCMV-JR-CSF*env* (B-SI V1–V3). Media was changed to DMEM + 2% FBS, 5–8 hours post transfection and supernatants were collected 48 hours later and frozen in 500 µl aliquots at −80°C until time of infection.

### Viral inactivation studies

Virus or pseudotyped viral vectors in media without serum, sufficient for an MOI of 0.1 were incubated on ice with or without 10 µg/ml defensin for one hour, then the defensins were removed by centrifugation over a Sephadex G-25 (Sigma, St. Louis, MO) column. Sephadex G-25 was prepared according to the manufacturer's protocol. Eluted virus was then spinfected onto GHOST-R5X4 cells. Some GHOST cells were incubated for one hour with defensins at 37°C prior to spinfection with virus [48]. Thirty-six hours post infection, the GHOST cells were harvested and GFP expression was analyzed by flow cytometry. For HNP-1 and HBD-2 the same procedure was done in activated PBMC with NL4-3 and JR-CSF viruses. Supernatants were collected seventy-two hours later and analyzed by p24 ELISA. All viral vector infections were performed in GHOST-R5X4 cells.
